# Supplementary material for: Mechanisms governing the pioneering and redistribution capabilities of the non-classical pioneer PU.1
Source: Nat Commun. 2020 Jan 21;11:402. doi: 10.1038/s41467-019-13960-2 (PMC6972792; doi:10.1038/s41467-019-13960-2)
Supplement: Supplementary file 7 — Source data [file 41467_2019_13960_MOESM7_ESM.zip › Source_Data/FigureS2/FigureS2B_MotifScanOutput/KmeansCluster4/homerResults/motif6.similar.html]

motif6

## Information for motif6

T
C
G
A
C
T
G
A
C
T
G
A
C
G
T
A
C
G
T
A
T
C
A
G
A
T
G
C
C
G
T
A
A
T
C
G
G
T
C
A
C
G
T
A
T
A
C
G
G
A
C
T
  
Reverse Opposite:  

C
T
G
A
A
T
G
C
G
C
A
T
A
C
G
T
A
T
G
C
A
C
G
T
T
A
C
G
A
G
T
C
G
C
A
T
G
C
A
T
G
A
C
T
G
A
C
T
A
G
C
T
  

|  |  |
| --- | --- |
| p-value: | 1e-186 |
| log p-value: | -4.286e+02 |
| Information Content per bp: | 1.665 |
| Number of Target Sequences with motif | 617.0 |
| Percentage of Target Sequences with motif | 13.67% |
| Number of Background Sequences with motif | 1187.1 |
| Percentage of Background Sequences with motif | 2.84% |
| Average Position of motif in Targets | 103.1 +/- 40.6bp |
| Average Position of motif in Background | 98.3 +/- 75.5bp |
| Strand Bias (log2 ratio + to - strand density) | 0.1 |
| Multiplicity (# of sites on avg that occur together) | 1.05 |
| Motif File: | file (matrix) reverse opposite |

### Similar de novo motifs found

|  |  |  |  |  |  |  |  |
| --- | --- | --- | --- | --- | --- | --- | --- |
| Rank | Match Score | Redundant Motif | P-value | log P-value | % of Targets | % of Background | Motif file |
| 1 | 0.934 | C G T A C G T A C G T A C T A G A G T C C G T A A C T G C G T A C G T A T A C G C G A T T A C G | 1e-124 | -287.310579 | 6.84% | 0.95% | motif file (matrix) |
| 2 | 0.953 | T C G A T C G A T C G A C G T A C G T A T C A G A G T C C G T A A C T G C G T A C G T A T A C G C G A T T A C G | 1e-118 | -273.974275 | 4.14% | 0.22% | motif file (matrix) |
| 3 | 0.859 | G C T A C G A T C G T A C T A G G A T C C G T A T A C G C G T A C G T A T A C G A G C T | 1e-109 | -252.340153 | 9.26% | 2.19% | motif file (matrix) |
| 4 | 0.792 | C T G A C G A T C A T G A T G C C G T A C A T G T G C A G T C A A T G C G A T C A T C G | 1e-41 | -95.643752 | 2.97% | 0.58% | motif file (matrix) |
| 5 | 0.797 | A G T C C G T A A C T G C G T A C G T A A C T G A C G T | 1e-37 | -85.540919 | 9.44% | 4.59% | motif file (matrix) |
